# IL-13 is a central mediator of chemical-induced airway hyperreactivity in mice

**DOI:** 10.1371/journal.pone.0180690

**Published:** 2017-07-13

**Authors:** Fien C. Devos, Lore Pollaris, Jonathan Cremer, Sven Seys, Tomoaki Hoshino, Jan Ceuppens, Karel Talavera, Benoit Nemery, Peter H. M. Hoet, Jeroen A. J. Vanoirbeek

**Affiliations:** 1 Centre for Environment and Health, Department of Public Health and Primary Care, University of Leuven, Leuven, Belgium; 2 Laboratory of Clinical Immunology, Department of Microbiology and Immunology, University of Leuven, Leuven, Belgium; 3 Division of Respirology, Neurology and Rheumatology, Department of Medicine 1, Kurume University School of Medicine, Kurume, Japan; 4 Laboratory of Ion Channel Research, Department of Cellular and Molecular Medicine, University of Leuven, Leuven, Belgium; Centre National de la Recherche Scientifique, FRANCE

## Abstract

**Background:**

While the importance of the Th2 cytokine IL-13 as a central mediator of airway hyperreactivity (AHR) has been described in allergic protein-induced asthma, this has never been investigated in chemical-induced asthma.

**Objective:**

We examined the importance of IL-13 in a mouse model of chemical-induced AHR, using toluene-2,4-diisocyanate (TDI).

**Methods:**

In a first set-up, wild type (WT) and *IL-13* knockout (KO) C57Bl/6 mice were dermally treated on days 1 and 8 with 1% TDI or vehicle (acetone/olive oil) on both ears. On day 15, mice received an intranasal instillation with 0.1% TDI or vehicle. In a second set-up, WT mice sensitized with 1% TDI or vehicle, received i.v. either anti-IL-13 or control antibody prior to the intranasal challenge.

**Results:**

TDI-sensitized and TDI-challenged WT mice showed AHR to methacholine, in contrast to TDI-sensitized and TDI-challenged *IL-13* KO mice, which also showed lower levels of total serum IgE. TDI-sensitized and TDI-challenged *IL-13* KO mice had lower numbers of T-cells in the auricular lymph nodes. TDI-treated WT mice, receiving anti-IL-13, showed no AHR, in contrast to those receiving control antibody, despite increased levels of IgE. Anti-IL-13 treatment in TDI-treated WT mice resulted in lower levels of serum IL-13, but did not induce changes in T- and B-cell numbers, and in the cytokine production profile.

**Conclusion and clinical relevance:**

We conclude that IL-13 plays a critical role in the effector phase of chemical-induced, immune-mediated AHR. This implicates that anti-IL-13 treatment could have a beneficial effect in patients with this asthma phenotype.

## Introduction

Asthma is a chronic airway disease, that encompasses many diverse phenotypes [1]. The most common and well-characterized phenotype is allergic (atopic) asthma [2]. This form of asthma is associated with T-helper (Th) 2-biased immune responses, resulting in the formation of allergen-specific IgE antibodies and release of Th2 cytokines [3,4]. Abundant evidence from both human and animal studies has shown that the Th2 cytokine IL-13 plays a central role in directing the immune response to an allergic asthma phenotype [5]. On its own, IL-13 is sufficient to induce some of the main characteristics of allergic asthma, i.e. airway hyperreactivity (AHR), airway inflammation via eosinophil recruitment, mucus production by airway epithelial cells and sub-epithelial fibrosis [5,6].

The prototypical type 2 cytokine, IL-13 is mainly produced by Th2 cells and innate lymphoid 2 cells, but can also be produced by many other immune cells, including Th1 cells, natural killer T-cells, mast cells, basophils and eosinophils [7,8]. The immunoregulatory function of IL-13 is mediated by its binding to a heterodimeric receptor complex, comprising IL-4 receptor α and IL-13 receptor α1, resulting in the activation of the signal transducer and activator of transcription (STAT)6 pathway, or by binding to an IL-13 specific chain, IL-13 receptor α2 [9].

The importance of IL-13 in regulating the pathogenesis of asthma in humans was demonstrated by genome-wide association studies, showing an association between polymorphisms of *IL-13* and its receptor and asthma susceptibility [2]. Patients with mild atopic asthma show an increased expression of *IL-13* in bronchoalveolar lavage (BAL) fluid and cells compared to control subjects [2]. In patients with this asthma phenotype, lebrikizumab, a monoclonal antibody to IL-13 is effective in improving lung function [10].

In murine models of allergic asthma, using ovalbumin as a classical protein allergen, IL-13 has been implicated as an inducer of AHR [11,12]. This was proven by using mice deficient in *IL-13* and wild type mice treated with anti-IL-13 monoclonal antibodies. Both set-ups resulted in a reduced AHR in response to an ovalbumin challenge [8,13,14]. In addition, it has been shown that AHR is lowered in *STAT6* deficient mice [15].

The importance of IL-13 in classic allergic asthma has thus been identified in both human and animal studies. However, many subtypes of asthma are distinct from ‘classic’ asthma, in which inflammation is mainly characterized by eosinophils. One of these subtypes is paucigranulocytic asthma, representing a substantial fraction of all asthma cases, and which can be induced by reactive chemicals [16,17]. In addition, up to 50% of all asthma cases are not attributable to atopy [18].

In mouse models of asthma induced by diisocyanates, a group of known chemical asthmogens, it has been shown that both Th2 and Th1 cytokines, including IL-13, IL-4 and IFN-γ, are associated with the development of asthma features. Depending on the mouse strain used and the airway challenge technique (aerosol, intranasal or oropharyngeal), an influx of lymphocytes, eosinophils and neutrophils into the lungs was accompanying airway hyperreactivity [19–21]. Yet, in a recently developed mouse model of non-atopic paucigranulocytic asthma, with increased serum IL-13 levels, the role of IL-13 remains largely unknown. To address this, we have used this validated mouse model of chemical-induced immune-mediated paucigranulocytic asthma, using TDI [22].

## Methods

### Reagents

Toluene-2,4-diisocyanate (TDI) (98%; Fluka, CAS 584-84-9), acetyl-β-methylcholine (methacholine) and acetone were obtained from Sigma-Aldrich (Bornem, Belgium). Pentobarbital (Nembutal®) was obtained from Sanofi Santé Animale (CEVA, Brussels, Belgium). Anti-mouse IL-13 antibody (262A-5-1) and mouse IgG1 anti-human gp120 were kindly provided by Genentech (San Francisco, California, USA). The vehicle (acetone/olive oil, AOO), used to dissolve TDI consisted of a mixture of 2 volumes of acetone and 3 volumes of olive oil (Selección de Almazara, Carbonell, Madrid, Spain) for both the dermal sensitization and the intranasal challenge. Concentrations of TDI are given as percent (v/v) in AOO.

### Mice

Male wild type C57Bl/6 mice (6–8 weeks old) were obtained from Harlan (Horst, The Netherlands). *IL-13* deficient mice on a C57Bl/6 background (8–10 weeks old) were kindly provided by Prof. Tomoaki Hoshino. All mice were housed in filter top cages in a conventional animal house with 12 h dark/light cycles, and they received lightly acidified water and pelleted food ad libitum.

### Experimental protocols of mouse experiments

As previously described, our model of chemical-induced asthma is based on prior systemic sensitization, induced by dermal applications of the test chemical, followed by an airway challenge. Non-specific airway hyperreactivity to methacholine, lung inflammation and immunologic responses are assessed one day later [22].

This protocol was applied to both wild type (WT) and *IL-13* knock-out (KO) mice. On days 1 and 8, mice were dermally treated with 1% of toluene-2,4-diisocyanate (TDI) or vehicle (acetone/olive oil, AOO, ratio 2:3) on the dorsum of both ears (20 μL/ear). On day 15, mice received an intranasal instillation of 30 μL of 0.1% TDI (challenge) or vehicle (AOO, ratio 2:3). Mice were sacrificed 24 h after the last challenge. Experimental treatment groups are referred to as AOO/AOO, AOO/TDI and TDI/TDI. The first symbol identifies the agent used for dermal applications on days 1 and 8 (sensitization), whereas the second symbol identifies the agent administered via intranasal instillation on day 15 (challenge). Experiments with the IL-13 KO mice were performed over a two-month period, during which we included mice depending on the availability of mice in successive litters. With each new litter, the available mice were distributed over the three treatment groups, with an aim to include six mice per group. The last mouse of the AOO/TDI groups did not survive the intranasal challenge and we decided not to include another mouse (n = 5). Some extra mice were sensitized with TDI and following a successful challenge included in the study. Therefore, the TDI/TDI groups has an n of 8.

The same sensitization protocol (treatment on days 1 and 8) was used to test if the specific anti-IL-13 antibody, 262A-5-1 influences the effector phase: thus, on days 13, 14 and 15, mice received either 2.5 mg/mL of mouse IgG1 anti-mouse IL-13 antibody, or mouse IgG1 anti-human gp120, used as control antibody, by intravenous (i.v.) injection (100 μL) (8). On day 15, 1 h after the last i.v. injection, mice received an intranasal challenge with TDI. Experimental groups are represented by three abbreviations: AOO/gp120/AOO, AOO/anti-IL-13/AOO, AOO/gp120/TDI, AOO/anti-IL-13/TDI, TDI/gp120/TDI and TDI/anti-IL-13/TDI. The first abbreviation identifies the agent used for sensitization, the second identifies the antibody that was injected i.v., and the third identifies the agent used for the airway challenge. All treatment groups contain eight mice. The analysis were spread-out over six experimental days, with each day two mice of each group.

### Non-specific airway hyperreactivity measurements

Twenty-four hours after the challenge, airway reactivity to methacholine was measured using a forced oscillation technique (flexiVent 7, SCIREQ, Montreal, Canada), as described previously [23]. Mice were anaesthetized by an intraperitoneal (i.p.) injection of pentobarbital (70 mg/kg body weight, Nembutal®, Sanofi Santé Animale, CEVA, Brussels, Belgium). The trachea was exposed and a 19-gauge metal needle was inserted. Mice were quasi-sinusoidally ventilated with a tidal volume of 10 mL/kg at a frequency of 150 breaths/min and a positive end-expiratory pressure of 3 cm H_2_O, to mimic the characteristics of spontaneous breathing. Airway resistance (R_n_) was measured using the ‘quick-prime 3’ protocol, which induces oscillations of 1 to 20.5 Hz during 3 seconds. After baseline measurements, each mouse was exposed to a methacholine aerosol, generated with an in-line nebulizer and administered at increasing concentrations (0, 1.25, 2.5, 5, 10, 20 mg/mL), each during 5 seconds. For each mouse, R_n_ was plotted against methacholine concentration and the area under the curve (AUC) was calculated to obtain a single measure of AHR and to perform the statistical analysis.

### Serum IgE

After measuring airway hyperreactivity to methacholine, mice were sacrificed. Blood was taken from the retro-orbital plexus, centrifuged (14000 g, 4°C, 10 min) and serum samples were stored at −80°C. The OptEIA^TM^ mouse IgE set from Pharmingen (BD Biosciences, Erembodegem, Belgium) was used to measure total serum IgE (diluted 1/70).

### IL-13 levels in serum and lung tissue homogenates

The Quantikine mouse IL-13 ELISA from R&D systems (Abingdon, UK) was used to measure IL-13 levels in serum and lung tissue homogenates. A part of the left lung was snap-frozen in liquid nitrogen and homogenized in a 5% BSA solution, using an Ultra-Turrax T25 (Ika Works, Staufen, Germany). Afterwards, homogenates were centrifuged at 4°C (1200 g, 10 min) and the supernatant was stored at −80°C. The ELISA was performed according to the manufacturer’s instructions, using undiluted serum samples or lung tissue homogenates. The detection limit was 1.5 pg/mL.

### Bronchoalveolar lavage (BAL)

The lungs were lavaged three times with 0.7 mL sterile saline (0.9% NaCl) in situ, and the recovered fluid was pooled. Cells were counted using a Bürker hemocytometer (total cell count) and the bronchoalveolar lavage (BAL) fluid was centrifuged (1000 g, 10 min). The supernatant was frozen (-80°C) until further analyses. For differential cell counts, 250 μL of the resuspended cells (100,000 cells/mL) were spun (300 g, 6 min) (Cytospin 3, Shandon, TechGen, Zellik, Belgium) onto microscope slides, air-dried and stained (Diff-Quik® method, Medical Diagnostics, Düdingen, Germany). For each sample, 200 cells were counted to determine the number of macrophages, eosinophils, neutrophils and lymphocytes.

### Lymph node analysis

Retro-auricular lymph nodes, obtained from the same mice were pooled and kept on ice in RPMI-1640 (Invitrogen, Merelbeke, Belgium). Cell suspensions were obtained by pressing the lymph nodes through a cell strainer (100 μm) (BD Bioscience, Erembodegem, Belgium) and rinsing with 10 mL tissue culture medium (RPMI-1640). After centrifugation (1000 g, 10 min), cells were counted using a Bürker hemocytometer and resuspended (10^7^ cells/mL) in complete tissue culture medium (RPMI-1640 supplemented with 10% heat-inactivated fetal bovine serum, 10 mg/mL streptomycin/penicillin). Five-hundred thousand cells were stained with anti-CD3^+^ (APC), anti-CD4^+^ (APC-Cy7), anti-CD8^+^ (PerCP-Cy5.5) and anti-CD25^+^ (PE), or received a single staining with anti-CD19^+^ (PE) labeled antibodies, according to standard procedures (BD Biosciences, Erembodegem, Belgium). Percentages of labeled cells were determined by performing flow cytometry (Facsarray, BD Biosciences, Erembodegem, Belgium) on at least 10^5^ cells.

Cells were seeded into 48-well culture plates at a density of 10^6^ cells/mL and incubated in complete RPMI-1640 medium for 42 h with 2.5 μg/mL of concanavalin A (ConA) (Sigma–Aldrich, Bornem, Belgium). Cell suspensions were then centrifuged (1000 g, 10 min) and supernatant was stored at −80°C. Levels of IL-4, IL-10, IL-13 and interferon gamma (IFN-γ) were measured in undiluted supernatant, via Cytometric Bead Array and analyzed with the FCAP Array Software (BD Biosciences, Erembodegem, Belgium) on the LSR Fortessa (BD Biosciences, Erembodegem, Belgium). The detection limits for IL-4, IL-10, IL-13 and IFN-γ were 0.3 pg/mL, 9.6 pg/mL, 2.4 pg/mL and 0.5 pg/mL, respectively.

### Data analyses

Dose-response curves (AHR) were analyzed using two-way parametric ANOVA, followed by a Bonferroni multiple comparison *post hoc* test. For all other data, normality of distribution was assessed by the Kolmogorov-Smirnov test. This data is presented as means with standard deviation (SD), and were analyzed using one-way parametric ANOVA, followed by a Bonferroni multiple comparison *post hoc* test (Graph Pad Prism 5. Graphpad Software Inc, San Diego, USA). A level of p < 0.05 (two-tailed) was considered to be significant.

### Study approval

All experimental procedures performed in mice were approved by the local Ethical Committee for animal experiments (P166-2012).

## Results

### Airway and immune responses in IL-13 KO mice

**Fig 1:**
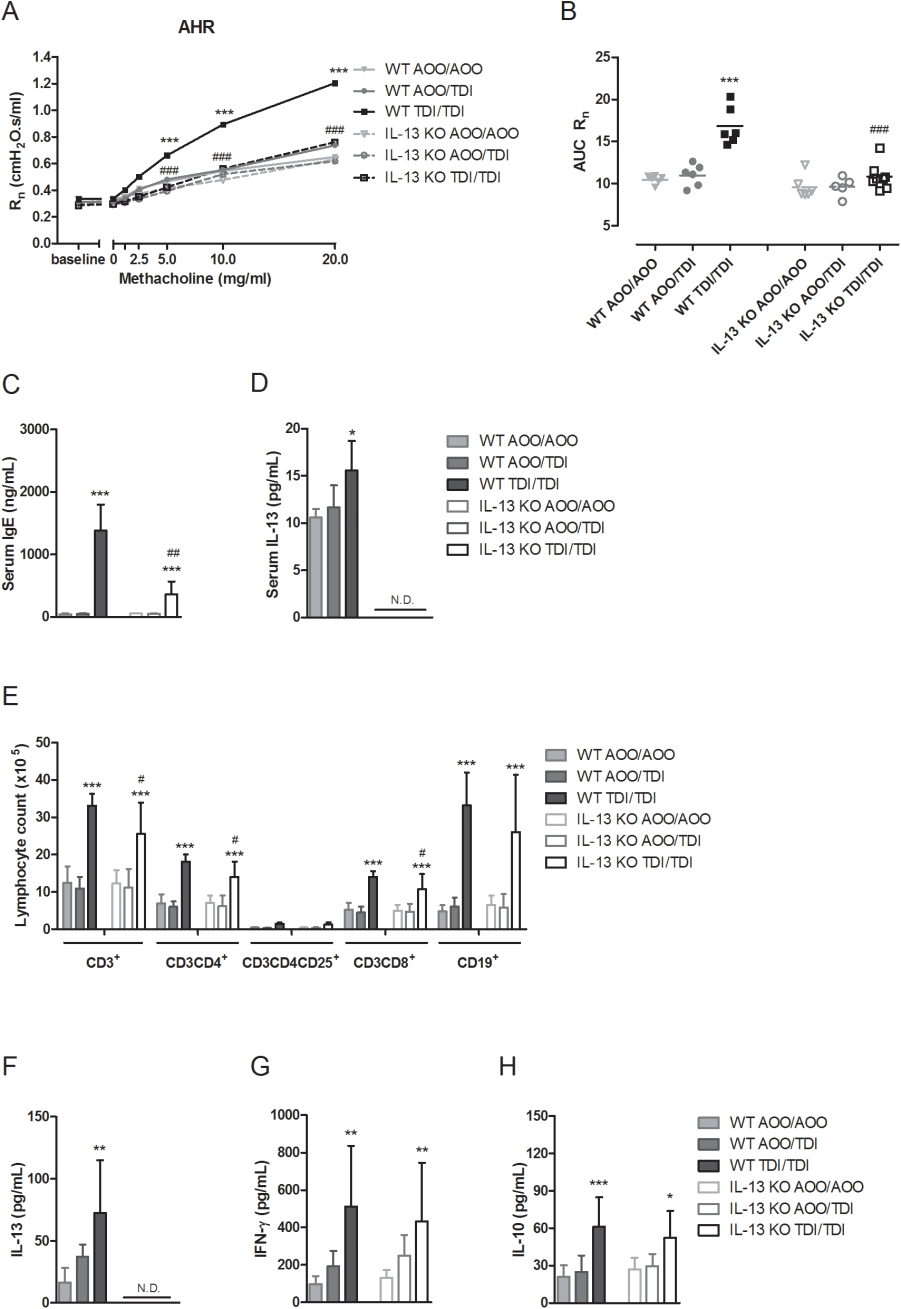
Airway and immune responses in *IL-13* KO mice. (A) Dose-response curves of R_n_ to methacholine (0–20 mg/mL). (B) Individual values and group means of the corresponding area under the curve (AUC) of airway hyperreactivity. (C) Total serum immunoglobulin E (IgE) and (D) IL-13 levels. (E) Lymphocyte subpopulations from auricular lymph nodes, stained with anti-CD3^+^, anti-CD3^+^CD4^+^, anti-CD3^+^CD4^+^CD25^+^ and anti-CD3^+^CD8^+^ or with anti-CD19^+^. Associated cytokine release of (F) IL-13, (G) IFN-γ and (H) IL-10. Data are presented as mean ± SD. * p < 0.05, ** p < 0.01 and *** p < 0.001 compared to own corresponding control group (WT AOO/AOO or *IL-13* KO AOO/AOO group). ^#^ p < 0.05, ^##^ p < 0.01 and ^###^ p < 0.001 compared to WT TDI/TDI group. N.D., non-detectable levels. n = 5–8 per group.

To examine the role of IL-13 in the development of sensitizer-induced AHR, responses to methacholine were measured in *IL-13* KO mice sensitized and challenged with TDI and compared to those of similarly treated WT mice. TDI-sensitized and TDI-challenged WT mice showed AHR, while this was not the case in *IL-13* KO mice. Airway resistance (R_n_) to methacholine (5–20 mg/mL) in TDI-treated *IL-13* KO mice did not differ from control *IL-13* KO mice, indicating that they were responsive to methacholine (Fig 1A and 1B). Cellular airway inflammation was absent in the BAL fluid of both WT and *IL-13* KO mice, confirming the paucigranulocytic phenotype of this model of chemical-induced asthma [22] (S1 Table).

Immune sensitization to TDI was investigated by assessing total serum IgE levels, IL-13 levels in serum and lung homogenates, and by assessing the lymph node subpopulations of the auricular lymph nodes, i.e. the draining site of sensitization, and the associated *ex vivo* cytokine release. WT mice sensitized and challenged with TDI showed significantly increased levels of total serum IgE and serum IL-13, compared to controls, whereas *IL-13* KO mice sensitized and challenged with TDI did not show detectable levels of serum IL-13 (Fig 1C and 1D). The TDI treated *IL-13* KO mice still showed significantly higher total serum IgE levels, yet, lower then TDI treated WT mice. No detectable levels of IL-13 were found in lung homogenates of both WT and *IL-13* KO mice. *IL-13* KO mice had similar lymphocyte subpopulations in the auricular lymph nodes, draining the site of sensitization, compared to WT mice. *IL-13* KO mice sensitized and challenged with TDI showed a significant increase in total number of CD3^+^ T-cells, CD4^+^ T-helper (Th)-cells, CD25^+^ activated and regulatory T (Treg)-cells, CD8^+^ cytotoxic T (Tc)-cells and CD19^+^ B-cells (Fig 1E). However, compared to WT mice, total numbers of CD3^+^ T-cells, CD4^+^ Th-cells and CD8^+^ Tc-cells were significantly lower in *IL-13* KO mice. In WT mice, this increase in lymphocyte numbers was accompanied by an increased release of Th2 (IL-13, IL-10) and Th1 (IFN-γ) cytokines, after *in vitro* stimulation of these lymphocytes with concanavalin A (ConA) (Fig 1F, 1G and 1H). As expected, *IL-13* KO mice showed no release of IL-13, yet releases of both IFN-γ and IL-10 in TDI-sensitized and challenged *IL-13* KO mice were significantly higher compared to the control treated *IL-13* KO mice and were not different from the WT mice receiving similar treatment (Fig 1F).

### Airway and immune responses in anti-IL-13 treated WT mice

**Fig 2:**
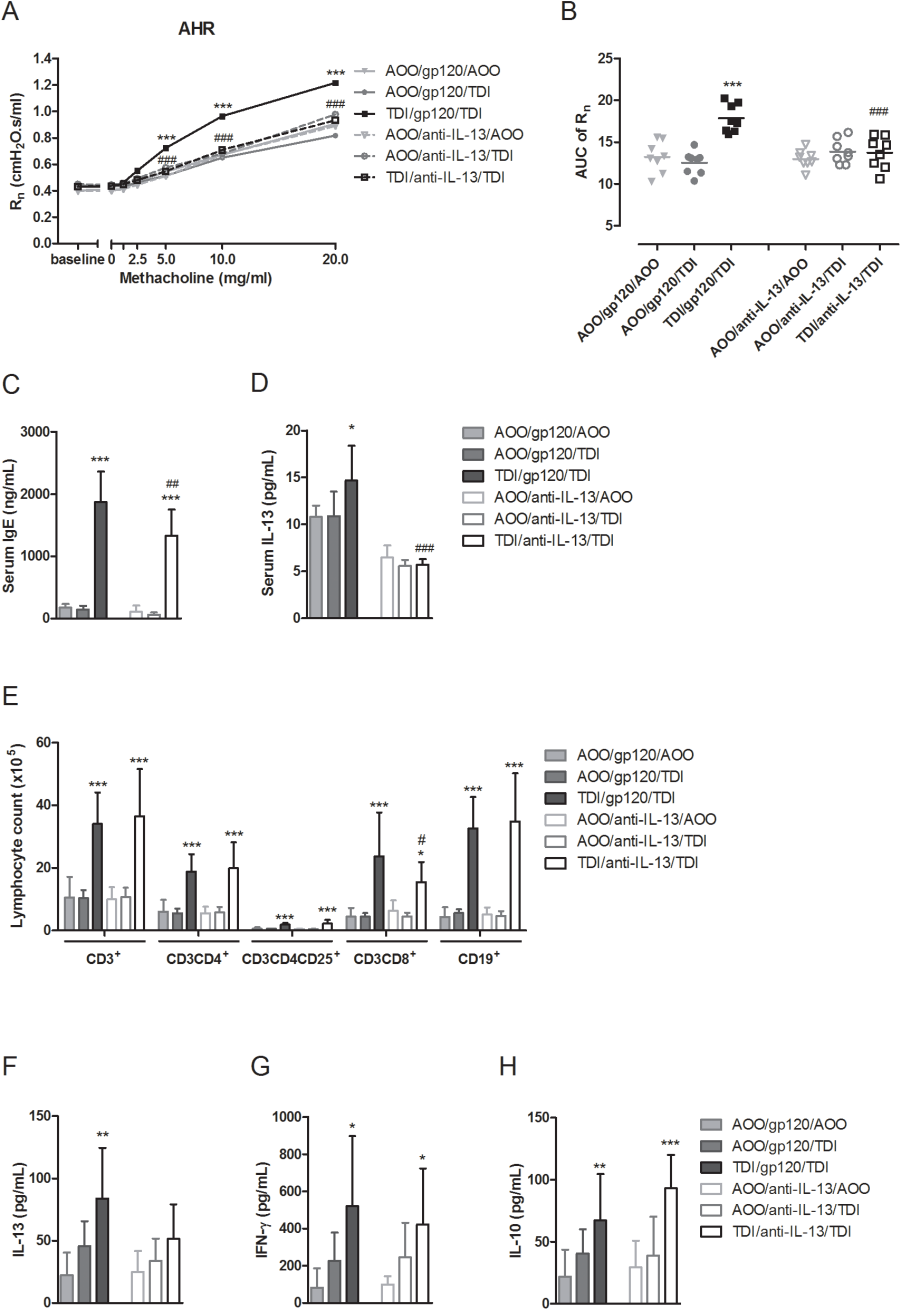
Airway and immune responses in anti-IL-13 treated WT mice. (A) Dose-response curves of R_n_ to methacholine (0–20 mg/mL). (B) Individual values and group means of corresponding area under the curve (AUC) of airway hyperreactivity. (C) Total serum immunoglobulin E (IgE) and (D) IL-13 levels. (E) Lymphocyte subpopulations from auricular lymph nodes, stained with anti-CD3^+^, anti-CD3^+^CD4^+^, anti-CD3^+^CD4^+^CD25^+^ and anti-CD3^+^CD8^+^ or with anti-CD19^+^. Associated cytokine release of (F) IL-13, (G) IFN-γ and (H) IL-10. Data are presented as mean ± SD. * p < 0.05, ** p < 0.01 and *** p < 0.001 compared to own corresponding control group (AOO/gp120/AOO or AOO/anti-IL-13/AOO), ^#^ p < 0.05, ^##^ p < 0.01 and ^###^ p < 0.001 compared to WT TDI/gp120/TDI group. n = 8 per group.

WT mice sensitized and challenged with TDI that received i.v. injections with anti-IL-13 did not develop AHR, in contrast to those that had been treated with the control antibody, gp120 (Fig 2A and 2B). Anti-IL-13 treatment significantly decreased R_n_ at methacholine doses from 5 to 20 mg/mL in mice sensitized and challenged with TDI, compared to control antibody treatment. Again, these mice did not show any inflammatory cells in the BAL fluid (S1 Table).

Total serum IgE levels were significantly increased in WT mice sensitized and challenged with TDI, treated with anti-IL-13 antibody, compared to controls (Fig 2C), although lower compared to those treated with the control antibody. Serum IL-13 levels remained at control levels in WT mice sensitized and challenged with TDI, treated with anti-IL-13 antibody, and were significantly lower than those treated with control antibody (Fig 2D). Lung homogenates of WT mice treated with anti-IL-13 or control antibody showed no detectable levels of IL-13.

Lymphocyte subpopulations in the auricular lymph nodes, site of sensitization, were not altered by anti-IL-13 treatment. Total numbers of CD3^+^ T-cells, CD4^+^ T-helper (Th)-cells, CD25^+^ activated and regulatory T-cells, CD8^+^ cytotoxic T (Tc) cells and CD19^+^ B-cells were significantly increased in TDI-sensitized and TDI-challenged mice, treated with either anti-IL-13 or control antibody, in comparison to the corresponding controls (Fig 2E). Total number of CD8^+^ Tc-cells was, however, significantly lower in mice sensitized and challenged with TDI, treated with anti-IL-13, compared to those treated with control antibody. The levels of *ex vivo* release of IL-13 in the supernatants of ConA-stimulated lymphocytes of TDI-sensitized and challenged mice WT mice, treated with IL-13 showed a higher trend, but was not significantly different from its untreated control (Fig 2F). For *ex vivo* release of IFN-γ and IL-10, significant increased levels were measured in the supernatant of lymphocytes from TDI-sensitized and challenged mice for both the gp120 control treated, as the anti-IL-13 treated group. (Fig 2G and 2H).

## Discussion

We have demonstrated that IL-13 plays an important role in chemical-induced AHR. In contrast to WT mice, TDI-sensitized *IL-13* KO mice failed to develop AHR 24 h after an airway challenge with TDI. The lack of IL-13 was accompanied by lower total serum IgE levels, lower numbers of T-cells, yet it did not affect the pro-inflammatory cytokines IFN-γ and IL-10. Still, numbers of T-helper cells, cytotoxic T-cells and B-cells were significantly increased in *IL-13* KO mice sensitized and challenged with TDI, compared to control *IL-13* KO mice.

Since IL-13 plays a role in both the sensitization and the effector phase of of classical asthma [6], the data obtained from the *IL-13* KO mice, hence reduced T-lymphocyte numbers and lower concentrations of systemic IgE, cannot fully explain the role of IL-13 in the effector phase. Therefore, we investigated the effect of IL-13 neutralization in WT mice sensitized to TDI, by using a specific anti-IL-13 antibody, following a treatment protocol effective in neutralizing IL-13 and abrogating AHR, as shown by Hacha *et al*., using a model of ovalbumin-induced asthma [8]. With this set-up, neutralizing IL-13 after the induction of TDI sensitization, evidenced by significantly lower levels of serum IL-13, AHR was absent 24 h after an airway challenge with TDI, despite increased serum IgE levels, increased T-and B-cell numbers and increased cytokine releases of IFN-γ and IL-10. This indicates that the lower concentration of IgE and the decrease in T-cell numbers in *IL-13* KO mice can be considered as an inherent aspect of the gene-deficiency, thereby rendering *IL-13* KO mice a less good tool to investigate responses depending on two phases, i.e. sensitization and challenge.

The prophylactic treatment with anti-IL-13 provided evidence concerning the contribution of IL-13 in the effector phase of the AHR response. Using a model of paucigranulocytic asthma, we also demonstrate that AHR can be established without the presence of cellular inflammation in the airways, as was already reported in a model of ovalbumin-induced asthma [24]. So, as was shown previously that although IL-13 is able to promote the recruitment of inflammatory cells into the airways, this is not necessary for the induction of AHR (12). This concept was supported by demonstrating that IL-13-induced AHR is not affected in IL-5 and/or eotaxin-deficient mice, and mice pretreated with a granulocyte inhibitor [25,26]. Moreover, in line with our results, IL-13 is able to induce AHR, independently of T- and B-lymphocytes [27].

*In vitro* studies, using airway smooth muscle cells of humans and rodents, have shown that IL-13 increases the contractility of smooth muscle, via enhancing Ca^2+^ oscillations of airway smooth muscle cells, thus implying a direct stimulatory effect of IL-13 on airway smooth cells [28–30]. In addition, IL-13 was shown to increase the affinity of smooth muscle for acetylcholine released from cholinergic nerves [31]. More recently, by using transgenic mice selectively expressing the IL-4 receptor α only in smooth muscle, Perkins *et al*. and Kirstein *et al*. showed that smooth muscle activation by IL-13 is sufficient, but not essential, to induce AHR [32,33]. Indeed, IL-13 can also act on airway epithelial cells to induce AHR, as shown by the use of transgenic mice expressing STAT6 only in airway epithelial cells [34]. Changes in the properties of airway epithelial cells, such as cell enlargement, increased permeability, increased rigidity and mucus production, ultimately lead to airway narrowing and subsequent AHR [32,34,35]. As such, it has been suggested that the effects of IL-13 on airway responsiveness rely on the combined activation of both airway smooth muscle cells and airway epithelial cells [11]. Considering the fact that the IL-4 and IL-13 receptor is ubiquitously expressed, the effects of IL-13 might also be induced via stimulation of cell types other than airway smooth muscle and epithelial cells, such as macrophages, mast cells, dendritic cells. Activation of these cells could in turn stimulate airway smooth muscle cells, epithelial cells or cholinergic nerves via the production of inflammatory mediators [32,33].

In our model of chemical-induced asthma, we have previously established a role for T- and B-lymphocytes, with the associated release of both Th1- and Th2-cytokines, including IL-13 [36–38]. TDI-treated ‘severe combined immunodeficiency’ mice lacking mature lymphocytes, showed no respiratory, nor inflammatory responses [36]. Also, the adoptive transfer of small amounts of lymphocytes from TDI-sensitized mice could passively sensitize naive mice and induced asthma-like responses after a specific airway challenge, indicating the importance of T- and B-lymphocytes [39,40]. More recently, we have also shown the involvement of neuro-immune interactions, with a role for airway sensory nerves expressing the chemo-receptors transient receptor potential (TRP)A1 and TRPV1, lymphocytes and mast cells in our model of chemical-induced immune-mediated paucigranulocytic asthma [22]. Mice lacking either TRPA1 (*Trpa1*^*-/-*^), TRPV1 (*Trpv1*^*-/-*^*)*, mature lymphocytes (*Rag2*^*-/-*^) or mast cells (*Kit*^*Wsh/Wsh*^), showed an abrogated AHR response. We concluded the existence of an interplay between mast cells and airway sensory nerves. Stimulation of these nerves occurs via the direct activation of TRPA1 by the isocyanate moieties of TDI and via the indirect activation of TRPV1 by immune-related inflammatory mediators [22]. Rehman *et al*. suggested a link between IL-13 and activation of neurogenic pathways, as IL-13 induced the expression of TRPV1 in the murine lung [41]. Further research is necessary to explore the effects of IL-13 on the neurogenic system in developing AHR.

In conclusion, we found IL-13 to be critically involved in the development of chemical-induced asthma, as shown here by using *IL-13* KO mice, and more specifically in the effector phase as confirmed by anti- IL-13 antibody treatment.

## Supporting information

S1 Table Supplementary file. (XLSX)
